# Airway management in patients with maxillofacial trauma – A retrospective study of 177 cases

**DOI:** 10.4103/1658-354X.76476

**Published:** 2011

**Authors:** Chetan B. Raval, Mohd. Rashiduddin

**Affiliations:** *Department of Anesthesia, Al-Nahdha Hospital, Muscat, Oman*

**Keywords:** *Difficult airway*, *fiberoptic bronchoscopic intubation*, *maxillofacial injuries*, *tracheostomy*

## Abstract

**Background:**

Airway management in maxillofacial injuries presents with a unique set of problems. Compromised airway is still a challenge to the anesthesiologist in spite of all modalities available. Maxillofacial injuries are the result of high-velocity trauma arising from road traffic accidents, sport injuries, falls and gunshot wounds. Any flaw in airway management may lead to grave morbidity and mortality in prehospital or hospital settings and as well as for reconstruction of fractures subsequently.

**Methods:**

One hundred and seventy-seven patients of maxillofacial injuries, operated over a period of one and half years during July 2008 to December 2009 in Al-Nahdha hospital were reviewed. All patients were reviewed in depth with age related type of injury, etiology and techniques of difficult airway management.

**Results:**

The major etiology of injuries were road traffic accidents (67%) followed by sport (15%) and fall (15%). Majority of patients were young in the age group of 11-30 years (71 %). Fracture mandible (53%) was the most common injury, followed by fracture maxilla (21%), fracture zygoma (19%) and pan-facial fractures (6%). Maxillofacial injuries compromise mask ventilation and difficult airway due to facial fractures, tissue edema and deranged anatomy. Shared airway with the surgeon needs special attention due to restrictions imposed during surgery. Several methods available for securing the airway, both decision-making and performance, are important in such circumstances. Airway secured by nasal intubation with direct visualization of vocal cords was the most common (57%), followed by oral intubation (17%). Other methods like tracheostomy and blind nasal intubation was avoided by fiberoptic bronchoscopic nasal intubation in 26% of patients.

**Conclusion:**

The results of this study indicated that surgically securing the airway by tracheostomy should be revised compared to other available methods. In the era of rigid fixation of fractures and the possibility of leaving the patient without wiring an open mouth and alternative techniques like fiberoptic bronchoscopic intubation, it is unnecessary to carry out tracheostomy for securing the airway as frequently as in the past.

## INTRODUCTION

Trauma is probably the most serious of all the major health problems faced by the developed countries. In the developing world the impact is just as great but has not been as extensively studied. In Oman road traffic accidents and sports-related injuries are the commonest injuries responsible for majority of maxillofacial injury. Maxillofacial trauma assumes importance as it involves vital and nonvital organs, looks ghastly, may lead to massive hemorrhage and is potentially life-threatening.[1] The first priority in these patients is airway maintenance with cervical spine control and Advanced Trauma Life Support (ATLS) concept for patients who sustained life-threatening injuries.[2] Subsequent reconstruction and fixation of fractures demand efficient and safe airway management. Morbidity and mortality of in-hospital trauma patients often result from critical care errors related to airway and respiratory management. Gruen *et al*. studied 2,594 trauma mortality patients in order to identify patterns of errors contributing to inpatient deaths.[3] They found that failure to intubate, secure or protect the airway was the most common factor related to patient mortality. Single universal technique of intubation may not be favorable in all circumstances so timely, decisive and skillful management of the airway can often make the difference between life and death or between ability and disability in such situations.

## METHODS

In a retrospective review, one hundred and seventy-seven patients of either sex, admitted to Al-Nahdha Hospital of maxillofacial trauma, operated during July 2008 to December 2009 as emergency and elective basis were studied. All patients were operated for open reduction and internal fixation under general anesthesia. Time of reporting to hospital was variable because some of them were admitted directly and others had taken primary treatment at other referring hospitals. Head injury and other associated injuries had already been addressed before taking up for surgery.

In preanesthetic assessment, patients were evaluated for types and mechanism of injury. Injury to vital and nonvital organs was ruled out. Decision for selection of intubation technique was taken on the basis of airway examination, type of fracture, type of procedure and patient’s co-operation. Airway examination was done by mouth opening – Mallampatti classification, thyromental distance, mento-hyoid distance and neck movement. Nasal patency was checked for all patients. Fiberoptic intubation technique was selected for patients with restricted mouth opening of one finger or less. Informed consent was obtained from all patients after detail explanation of procedure and alternative technique.

All adult patients were premedicated with midazolam 7.5 mg orally 1 hour before surgery. Adult patients for fiberoptic procedure received IM pethidine 1 mg/kg, promethazine 0.5 mg/kg and atropine 0.1 mg/kg. Children were premedicated with midazolam 0.5 mg/kg orally 1 hour before the start of anesthesia. Glycopyrrolate 0.2 mg IV was given for those who had not received atropine.

In patients considered to have adequate airway, routine monitoring was used. Difficult intubation cart was kept ready. All children were induced with sevoflurane 8% in 100% oxygen via face mask. Adult patients in whom airway was considered adequate, general anesthesia was induced with thiopentone 3-5 mg/kg. Succinylcholine 2 mg/kg was used for intubation, and maintenance with nondepolarizing muscle relaxant atracurium and isoflurane/sevoflurane. Analgesia was supplemented with fentanyl 1-2 mcg/kg and IV paracetamol 1 g (15mg/kg in children) in titrated dose. Anesthesia was reversed at the end of surgery with Neostigmine 50 mcg/kg and atropine 20 mcg/kg administered intravenously. Extubation was carried out with smooth emergence and patients in fully awake state, breathing spontaneously, obeying commands and satisfactory muscle power. Postoperative analgesia provided with Diclofenac 1mg/kg IM 20 min before extubation. Postoperative care was given in the high dependency unit.

For adult patients in whom, airway was considered inadequate and fiberoptic-guided intubation under sedation was considered, topical anesthesia was achieved by nasal packing with lignocaine 4% and xylometazoline 0.1% nasal drops. Pharynx was sprayed with 4-6 puffs of lidocaine 10% aerosol. Tracheal mucosa was anesthetized through cricothyroid injection of 2 ml 2% lignocaine after aspiration of air and immediately before coughing. Sedation was supplemented with fentanyl i.v in titrated doses.

For children with compromised airway in whom fiberoptic-guided intubation was under consideration, inhalation induction of anesthesia was started with sevoflurane 8% in 100% oxygen via face mask. According to depth of anesthesia, inspiratory concentration of sevoflurane was reduced to 4-5 %. A nasal endotracheal tube (one size smaller than the predicted size) was lubricated well and inserted in the contralateral nostril as soon as depth of anesthesia was deemed appropriate. Length of the nasal tube was measured by traditional method: from tip of the nose to the external auditory meatus. This tube was used as a conduit through which inhalational anesthesia was maintained. Depth of anesthesia was assessed and was considered adequate for FOI when pupils were in neutral position, normal size and no eyeball activity noted, and no response on vigorous anterior displacement of the mandible. The bronchoscope was then inserted through other nostril.

In both adults and children, topical anesthesia of the larynx was applied via “Spray as-you-go” technique through the suction channel of the bronchoscope using injections of 2 ml of Lidocaine 4%. Advancement of the fiberscope was withheld for 1 min to allow for the drug’s anesthetic effect. Portex endotracheal tube was railroaded on to the well-lubricated Karl Storz Fibrebronchoscope. When the larynx was visualized, the endotracheal tube was advanced into the larynx till the carina was seen. The bronchoscope was removed and the tube was fixed after assuring correct tube placement by visualization of the chest movement, breath sounds and ETCO_2_ monitoring.

## RESULTS

One hundred and seventy-seven patients were operated at a tertiary care hospital for a period of one and half years during July 2008 to December 2009. In total of 177 patients [Table 1], 150 (85%) were male and 27 female (15 %).

**Table 1:** Age distribution

Age (years)	No. of patients (n= 177)
1 - 10	20 (11)
11 - 20	33 (19)
21 - 30	91 (52)
31 - 40	18 (10)
41 and above	15 (8)

Figures in parentheses are in percentage

Majority of patients were young in the age group of 11–30 years [124 (71 %)] that is because of road traffic accidents and sport injury. In the age group of 1-10 years, major etiology was fall. Other group’s etiology was mixed. The mean age of the patients was 24 with the range of 3-63 years. Overall the major etiology [Table 2] of maxillofacial trauma was road traffic accidents 120 patients (67%). Main sport in Oman is football, so sports injury was observed in 27 patients (15%). In 26 patients (15%), the etiology was of fall, among them children, older age and construction workers. Only 1 patient was a child had a gun-shot injury.

**Table 2:** Aetiology of trauma

Aetiology of trauma	No. of patients (n=177)
Road traffic accidents	120 (67)
Sports injury	27 (15)
Fall	26 (15)
Assault	3 (2)
Gunshot and blast injury of face	1 (1)

Figures in parentheses are in percentage

Fracture mandible [Table 3] was found to be the most common injury in 96 patients (53%) followed by fracture maxilla in 37 patients (21%) and Zygoma in 33 patients (19%). There was pan-facial fracture (more than two fractures of face) in 10 patients (6%).

**Table 3:** Type of maxillofacial trauma

Type of maxillofacial trauma	No. of patients (n=177)
Fracture mandible	96 (53)
Fracture maxilla	37 (21)
Fracture zygoma	33 (19)
Pan facial fracture	10 (6)
Gunshot and blast injury of face	1 (1)

Figures in parentheses are in percentage

According to airway status and familiarity of technique with respective anesthetist, nasal intubation with direct visualization of vocal cords was achieved in 100 patients (57%) [Table 4]. Oral intubation was done in 30 patients (17%), whenever permitted by maxillofacial surgeon according to type of surgery–mostly fracture zygoma. Patients considered having difficult airway, co-operation with procedure and familiarity of technique of respective anesthetist, fiberoptic-guided nasal intubation with sedation was done in 38 patients (21%). While in children and uncooperative nine patients (5%) with difficult airway, fiberoptic-guided intubation was done under GA with spontaneous respiration. These techniques had avoided tracheostomy, blind nasal intubation and submental intubation and its complications.

**Table 4:** Various techniques of intubation used during anesthesia

Various techniques of intubation used during anesthesia	No. of patients (n=177)
Nasal intubation	100 (57)
Oral intubation	30 (17)
Fibreoptic guided nasal intubation with GA	9 (5)
Fibreoptic guided nasal intubation with sedation	38 (21)
Tracheostomy	–
Blind nasal intubation	–
Sub-mental intubation	–

Figures in parentheses are in percentage, GA = General anesthesia

Other associated injuries and head injury had already been addressed before taking up for surgery.

## DISCUSSION

Trauma accounts for thousands of deaths and financial burden on any country. It has been labeled as “neglected disease of modern society”. It involves universal young productive lives and male predominance. For every death, two people suffer permanent disability. Maxillofacial injuries need special attention due to many reasons. These injuries are with or without head injury and cervical spine fractures or polytrauma. Early airway control requires sound judgment and considerable experience. Skillful experienced personnel are mandatory. In order to have a good outcome with minimal risks and maximal success in airway management, should be in collaboration with the anesthesiologist or trauma team leader is must.[1] ATLS protocol must be followed in all cases of maxillofacial trauma with immediate attention to life-threatening injuries.[2] Gruen *et al* found that, failure to intubate, secure or protect the airway was the most common factor related to patient mortality, responsible for 16% of inpatient deaths.[3]

Emergency trauma care was not part of this study since primary care was given separately. The time lag between the injury and surgery is variable depending on primary care institutional protocols and may range from few hours to few days according to associated injury, facial edema and preoperative optimization of general condition. Resolution of facial edema during this time allows for more accurate clinical evaluation of airway and ease of intubation. Capasi *et al*. suggested that the delay in final reconstruction of facial fractures in critically ill patient has an acceptably low complications rate and may be advantageous in decreasing operative risk.[4] Hutchinson *et al*. addressed six specific situations associated with maxillofacial trauma, which may adversely affect the airway: 1. Postero-inferior displacement of a fractured maxilla parallel to the inclined plane of the skull base, 2. bilateral fracture of the anterior mandible, 3. hemorrhage, 4. soft tissue swelling and edema, 5. trauma to the larynx and trachea, 6. foreign bodies – dentures, debris, shrapnel, exfoliated teeth, bone fragments.[5] Planned reconstruction schedule is required to achieve maximum, satisfactory function and appearance as unnecessary delay in surgery may predispose to complications like malunion and infections.

Approach to the maxillofacial trauma patient’s airway evaluation and preparation is the key to a successful anesthetic management. Extent of injury, the composition and the anatomy of the injury along with Mallampatti classification, atlantoaxial mobility and thyromental distance provides good airway assessment.[1] But these all may not be accurate in the presence of tissue edema, disrupted anatomy and muscle spasm. The risk of airway-related complications during the peri-operative period was studied by Peterson *et al*.[6] They analyzed the American Society of Anesthesiologists Closed Claims database to identify the patterns of liability associated with the management of the difficult airway. They found that complications arose throughout the peri-operative period: 67% upon induction, 15% during surgery, 12% at extubation and 5% during recovery. As with every difficult airway situation, the equipment for difficult intubation should be prepared and ready to use. The approach should be chosen according to the patient’s injuries, airway status and the care provider’s experience with such equipment and procedures.

Management of the airway is a major concern in patients with maxillofacial trauma (gunshot wounds, facial fractures, cervical spine injuries, laryngotracheal injuries) because a compromised airway can lead to death. The method of intubation to use in these patients remains a controversial topic. Although there are many options available, each one has specific indications, and the choice will ultimately depend on the patient’s situation and the expertise of the anesthesiologist.[7]

Flexible fiberoptic intubation under local anesthesia with sedation is the technique of choice for management of the anticipated difficult airway with restricted mouth opening and difficult mask ventilation in the patient undergoing an elective procedure.[8–10] Arrowsmith *et al* stated in their study that basal skull fracture should not be regarded as an absolute contraindication to nasotracheal intubation. It is absolutely safe to do nasotracheal intubation under vision by fiberoptic bronchoscope in basal skull fracture.[11] In maxillofacial trauma patient’s blood, vomitus and secretions in the patient’s airway preclude vision by fiberoptic instruments. In addition, accomplishing effective local anesthesia in the traumatized region is difficult. Furthermore, the patient’s cooperation is essential for such an approach. This needs perfection and patience on the part of anesthesiologist.[12,13] When the patient is an unco-operative child, flexible fiberoptic intubation is to be done under GA. Fiberoptic intubation of the spontaneously breathing patient is the gold standard and technique of choice for the elective management of a difficult airway. In the hands of the properly trained and experienced user, it is also an excellent alternative when direct laryngoscopy unexpectedly fails. Fiberscope-assisted intubation through an endoscopy face mask, laryngeal mask airway or intubating laryngeal mask airway secures ventilation and oxygenation, and permits endotracheal intubation in airway emergency situations.[14,15] But in maxillofacial patients all above devices fails due to facial edema, restricted mouth opening, so GA can be maintained through other means like simple ET tube in the other nostril[16,17] or nasopharyngeal airway.[18] Avoiding airway irritation and laryngeal spasm by using topical anesthesia in GA and it increases the success rate in awake technique. Maximum allowable dose of lignocaine is 4.5 mg/kg.[19] Our study showed that fiberoptic-guided nasal intubation with sedation was done in 38 patients (21%). While in children and uncooperative nine patients (5%) with difficult airway, fiberoptic-guided intubation was done under GA with spontaneous respiration. These techniques had avoided tracheostomy, blind nasal intubation and submental intubation and its complications.

Ovassapian *et al* suggested that blind nasal intubation is a simple technique and excellent alternative in the absence of fiberscope but it has two major drawbacks: infrequent success on the first pass and increased trauma with repeated attempts, precipitating complete airway obstruction that necessitates emergent cricothyrotomy.[20] Tracheostomy under local anesthesia had been considered gold standard of difficult airway management and for postoperative period.[21] Kearney *et al* found that tracheotomy by itself carries a 5% risk of complications, such as hemorrhage or pneumothorax.[22] Taicher *et al* study indicated that surgically securing the airway by tracheostomy should be revised compared to other available methods. In the era of rigid fixation of fractures and the possibility of leaving the patient with an open mouth, it is unnecessary to carry out tracheostomy for securing the airway as frequently as in the past.[23]

Maxillofacial surgeons prefer to have nasal intubation as it gives them freedom to operate and the accuracy of dental occlusion.[24] This requirement guided us to carry out nasal intubations in majority of cases. Oral route endotracheal intubation may thwart this accuracy of dental occlusion, so in our study it was used in zygoma fracture cases where occlusion is not needed [30 patients (17%)]. Nasal or oral intubations, with direct visualization of vocal cords and manual in-line stabilization in cases of cervical spine fractures dose not present much problem if there is no gross anatomical disruption. However there was no patient with basilar skull fracture. Dutta *et al* shown in that study that retromolar positioning of the tracheal tube in the retromolar trigone during intermaxillary fixation provides an optimal intraoperative control of dental occlusion.[25] The tube is to be fixed at the angle of the mouth in retromolar access. Extubation can be achieved from the retromolar space, when patient is awake. But Rungta *et al* stated that a wire cutter should always be kept ready beside the patient in case of emergency.[26,27] Submental and both anterior and lateral submandibular routes have been described in the surgical management of severe pan facial fractures. The technique, in its various forms is said to be relatively simple and safe to perform and produces a cosmetically acceptable scar.[7,26,28,29] In a recent review by Caubi *et al*, submental intubation has been found to be safe but observed increases in tracheal pressure as a result of deviation and compression of tube.[30] Since we did not have experience in these techniques it was not considered. Airtraq, Macintosh laryngoscopes, PAXpress and Frova single-use tracheal tube introducer like intubation aids have been tried with variable results recently.[31–33] Retrograde oral or nasal and submental intubation, utilizing epidural catheter may be other good alternative in difficult or failed intubations.[34,35]

Our figures are a reflection of the other studies. Road-traffic accidents [120 patients (67%)] are the major etiology in age group of 11–30 years [124 (71 %)] of which were male patients [150 (85%)].[36,37] But the major etiology in the age group of 1-10 years was fall from height as comparable to other studies.[38,39] Sport injuries [27 patients (15%)] producing the greatest number in young age group and it is the second etiology in our study. Sport related mandibular and zygomatic fractures were common as mentioned in other studies.[40–42] Fracture mandible was found to be the most common injury in 96 patients (53%), followed by fracture maxilla in 37 patients (21%) and zygoma in 33 patients (19%). This was comparable with the study of Motamadi *et al*.[43] The findings of this study, compared with similar studies reported in the literature, support the view that the causes and incidence of maxillofacial injuries vary from one country to another.

## CONCLUSION

This study dissected the distinct impact of injury mechanisms, types of fracture and age group in maxillofacial trauma in Oman. The results of this study indicated a considerable change in the airway management techniques and shows that surgically securing the airway by tracheostomy should be revised compared to other available methods. In the era of rigid fixation of fractures and the possibility of leaving the patient with an open mouth and alternative techniques like fiberoptic bronchoscopic intubation, it is unnecessary to carry out tracheostomy for securing the airway as frequently as in the past. Technique like fiberoptic, submental and submandibular intubations need more expertise, but can provide efficient airway control. It needs regular practice and learning sessions. It is important to remember that timely, decisive and skillful management of the airway can often make the difference between life and death or between ability and disability in such situations. Single universal technique of intubation may not be finding favorable in all circumstances.
